# New insight in the architecture of the quadriceps tendon

**DOI:** 10.1186/s40634-016-0068-y

**Published:** 2016-11-03

**Authors:** Karl Grob, Mirjana Manestar, Luis Filgueira, Timothy Ackland, Helen Gilbey, Markus S. Kuster

**Affiliations:** 1 Department of Orthopaedic Surgery, Rorschacher Strasse 95, CH-9007 St. Gallen, Switzerland; 2 Department of Anatomy, University of Zürich-Irchel, Winterthurerstrasse 190, CH-8057 Zürich, Switzerland; 3 Department of Anatomy, University of Fribourg, Rue Albert Gockel 1, CH-1700 Fribourg, Switzerland; 4 The University of Western Australia, 35 Stirling Highway, Crawley, WA 6009 Australia; 5 Hollywood Functional Rehabilitation Clinic, Perth, WA Australia

**Keywords:** Quadriceps tendon, Tensor vastus intermedius, Extensor apparatus of the knee joint, Quadriceps muscle group

## Abstract

**Background:**

Published data regarding the structure of the quadriceps tendon are diverse. Dissection of the quadriceps muscle group revealed that beside the rectus femoris, vastus lateralis, vastus intermedius and vastus medialis a fifth muscle component– named the tensor vastus intermedius consistently fused into quadriceps tendon. It can be hypothesized that all these elements of the extensor apparatus of the knee joint must also be represented in the quadriceps tendon. This study investigated the multi-layered quadriceps tendon with special emphasis on all components of the quadriceps muscle group including the newly discovered tensor vastus intermedius.

**Methods:**

Ten cadaveric lower limbs were dissected. All muscle bellies of the extensor apparatus of the knee joint were identified and traced distally until they merged into the quadriceps tendon. Connections between the different aponeurotic layers of each muscle were studied from origin to insertion. The fusing points of each layer were marked. Their distance to the patella and the distances between the fusing points were measured.

**Results:**

Six elements of the quadriceps muscle group form a tri-laminar structure of the quadriceps tendon. The intermediate layer could be further sub-divided. The elements of the quadriceps tendon are 1. lateral aponeurosis of the vastus intermedius, 2. deep and 3. superficial medial aponeurosis of the vastus intermedius, 4. vastus lateralis, 5. tensor vastus intermedius and 6. rectus femoris. Even with differences in fiber direction – these elements join each other a certain distance proximal to the patella.

All elements were fused over a region measuring 13 to 90 mm proximal to the patella. Lateral parts of the vastus intermedius formed the deepest layer of the quadriceps tendon. The superficial and deep layer of the medial vastus intermedius aponeurosis fused 56 mm (range, 30 to 90 mm) and 33 mm (range, 13 to 53 mm) above the patella with the aponeurosis of the tensor vastus intermedius and vastus lateralis respectively. Together they built the two-layered intermediate layer of the quadriceps tendon. The tendon of the rectus femoris forms the superficial layer. The vastus medialis inserts medially in all layers of the quadriceps tendon.

Fibers of the lateral muscle components were oriented towards the medial, and fibers of the medial muscle components were oriented towards the lateral femoral condyle.

**Conclusions:**

The three-layered quadriceps tendon is formed by six elements. These are 1. lateral aponeurosis of the vastus intermedius, 2. deep and 3. superficial medial aponeurosis of the vastus intermedius, 4. vastus lateralis, 5. tensor vastus intermedius and 6. rectus femoris. These elements of the extensor apparatus join each other proximal to the patella in a complex onion-like architecture. Its two-layered intermediate layer shows variable fusions points. The vastus medialis contributes to the quadriceps tendon with its medial insertion into all layers of the quadriceps tendon.

## Background

The insertion of the quadriceps femoris into the patella is traditionally described as a common tendon with a tri-laminar arrangement (Andrikoula et al. 2006; Iriuchishima et al. 2012; Sonin et al. 1995; Warwick & Williams 1973; Yablon et al. 2014), with the most superficial fibers originating from the rectus femoris, the deepest layer from the vastus intermedius and the intermediate layer from the vastus lateralis and vastus medialis. Other studies have suggested that the quadriceps tendon anatomy is more variable with a two- to four-layered or even more complex organisation, often with unequal contributions from its tendinous constituents (Waligora et al. 2009; Zeiss et al. 1992). Considering the consistent components of the quadriceps muscle group, the published variability of the tendon composition seems surprising.

In recent anatomical studies, an intervening muscle, between the vastus lateralis and vastus intermedius – named the tensor vastus intermedius - has been identified (Grob et al.; Rajasekaran & Hall 2016). Depending on the relation to the adjacent vastus lateralis and vastus intermedius muscles, different morphological types of tensor vastus intermedius were identified. The aponeurosis of the tensor vastus intermedius consistently fused into the middle layer of the quadriceps tendon and inserted at the superior medial border of the patella. As this muscle was previously attributed to other parts of the quadriceps muscle group, its role in the organization of the quadriceps tendon was unknown (Grob et al.).

A thorough understanding of the architecture of the knee extensor mechanism is of clinical importance. The quadriceps tendon is involved in many orthopaedic procedures around the knee joint including surgical approaches (Apostolopoulos et al. 2010; Koninckx et al. 2014; Rossi et al. 2014), tendon injuries (Rehman & Kovacs 2015) or harvesting as a tendon graft (Geib et al. 2009; Kim et al. 2009; Lund et al. 2014; Noyes & Albright 2006). Patellar problems are also common after total knee arthroplasty (Russell et al. 2014). A better understanding of the quadriceps tendon anatomy is therefore fundamental for an improvement in surgical techniques and for the radiological interpretation of a traumatized extensor apparatus of the knee joint (Yablon et al. 2014; Zeiss et al. 1992).

## Purpose and hypothesis

The newly described tensor vastus intermedius contributes to the extensor apparatus of the knee joint (Grob et al.; Rajasekaran & Hall 2016). It can be hypothesized that the tensor vastus intermedius as a fifth component of the quadriceps muscle group might represent a specific section in the in the quadriceps tendon. It has been shown, that the aponeurotic tendon fuses into the quadriceps tendon and inserts at the superior medial border of the patella (Grob et al.; Rajasekaran & Hall 2016). The purpose of the present study was to further investigate the multi-layered structure of the quadriceps tendon with special emphasis on on all components of the extensor apparatus.

## Methods

**Fig. 1:**
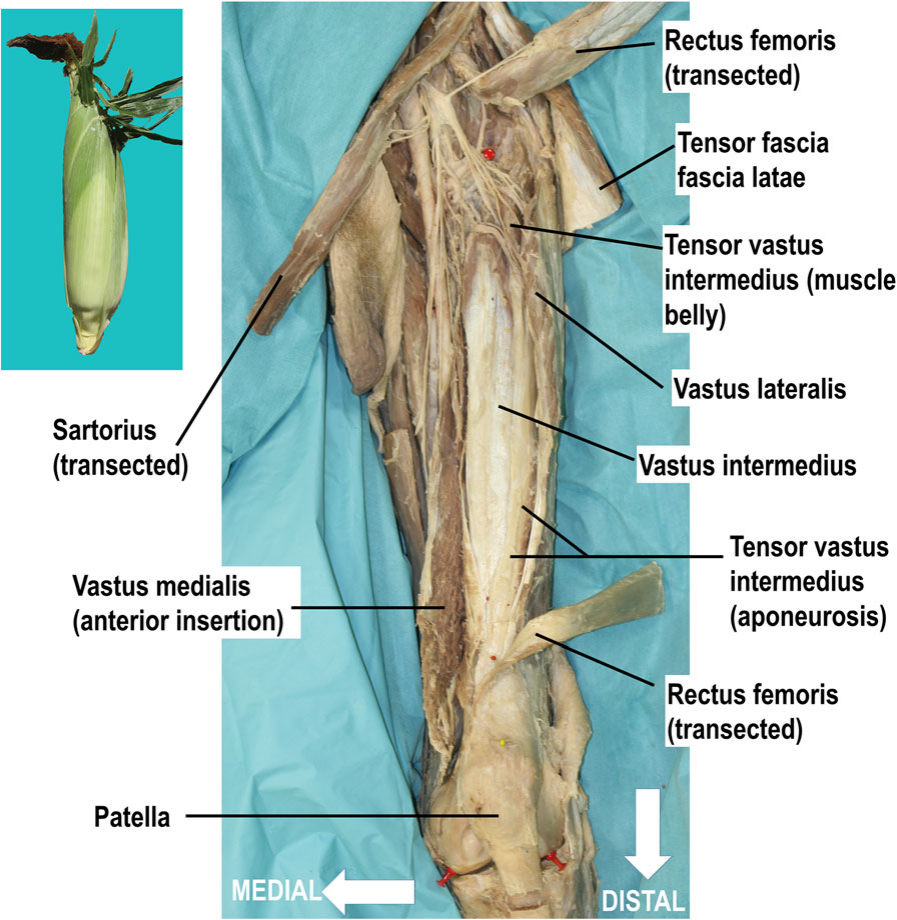
Overview of the quadriceps muscle group including the newly discovered fifth component, the tensor vastus intermedius. Anterior view to the left thigh is shown. The red stickers mark the medial and lateral femoral condyles and center of the neck of the femur respectively. For better visualization the sartorius and rectus femoris muscle are transected and reflected. The components of the extensor apparatus are arranged like the layers of a husk of a corn (*on the left top*). Superficial lateral and medial fibers in the proximal aspect of the thigh are piled in deeper inner layers at the level of the quadriceps tendon. The vastus medialis is released from its insertion into the vastus intermedius, rectus femoris and patella

Ten cadaveric lower limbs from 7 specimens, three paired and four unpaired (5 men and 2 women; mean age at death 78 years) were investigated using macro dissection techniques. The cadaver parts were obtained from the institutional body donation program (http://www.anatomy.uzh.ch/de/koerperspende.html) following the ethical guidelines “On the use of cadavers and parts of cadavers in medical research and for pre-, postgrad and continued education and research with human subjects” by the Academy of Medical Sciences (SAMS). All lower limbs were embalmed in a formalin-based solution. The thighs were examined on the basis of a standardized dissection protocol. Each lower limb was placed supine on the dissection table. The hip joint was approached from the anterior aspect and the tensor fasciae latae muscle mobilized laterally. The femoral nerve and artery were localized via a second ilio-inguinal approach, and traced distally. With the aid of these neurovascular structures, the muscle bellies of the rectus femoris, the vastus lateralis, the tensor vastus intermedius, vastus intermedius, and vastus medialis were identified. For better visualization the rectus femoris and sartorius were transsected in the mid portion and reflected. Each muscle with its aponeurosis was traced from proximal to distal until they merged into the quadriceps tendon. Connections between the different aponeurotic layers of each muscle were studied from origin to insertion (Fig. 1), with special emphasis on corresponding muscle fibers from the medial and lateral elements. The fusing points of each layer were marked. Their distance to the patella and the distances between the fusing points were measured.

## Results

All portions of the extensor apparatus fused over a region ranging from 13 to 90 mm (mean 44 mm, SD +/− 21) proximal to the superior pole of the patella medial to the mid-line of the quadriceps tendon. The different components were structured in onion-like layers or similar to a husk of a corn. Superficial lateral and medial fibers in the proximal aspect of the thigh were piled in deeper inner layers at the level of the quadriceps tendon (Fig. 1). The thickness of the quadriceps tendon increased steadily as more aponeurotic layers of the extensor apparatus joined both medially and laterally. At the patella insertion the quadriceps tendon reached its maximal thickness of 79 mm (range 65 to 95 mm, SD +/− 0.9). Deep to the quadriceps tendon between the tendon and the femur, the muscle bundles of the articularis genus extended to the suprapatellar bursa, and the synovial membrane of the knee joint. The fibers of the articularis genus did not contribute to the architecture of the quadriceps tendon, but merely fused with the dorsal side of the aponeurosis of the vastus intermedius.

**Fig. 2:**
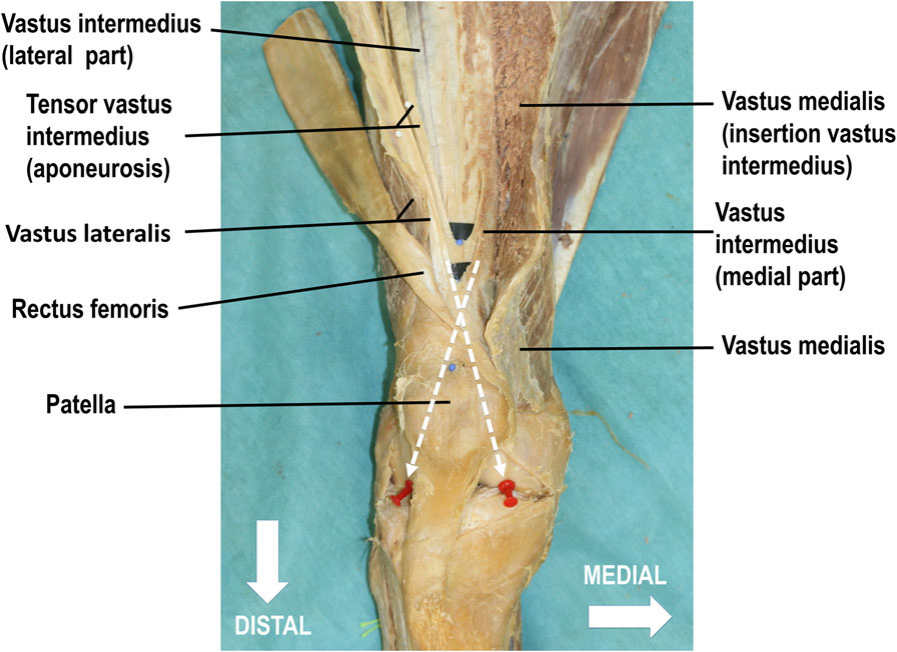
Orientation of the multilayered structure of the extensor apparatus of the knee joint. The distal aspect of a right thigh is shown. Red stickers mark the medial and lateral femoral condyles above the knee joint space. The vastus medialis is released from its insertion into the vastus intermedius and rectus femoris (*reflected laterally*) freeing the view to the complex multi-layered aponeurosis of the vastus intermedius. The lateral part of the vastus intermedius aponeurosis form the deepest layer of the quadriceps tendon. The medial part of the vastus intermedius aponeurosis separates into a superficial and deep medial layer with an orientation towards the lateral femoral condyle (*lateral red stick*). The fibers of the medial vastus intermedius aponeurosis are located above the lateral part of the vastus intermedius aponeurosis. Generally, lateral fibers are oriented towards the medial femoral condyle (*medial red stick*). The blue dots mark the fusing points of the intermediate layer of the quadriceps tendon and the superior base of the patella. For better visualization the fusing points are underlined with black paper. The white dotted lines indicate the fiber direction towards the condyles

**Fig. 3:**
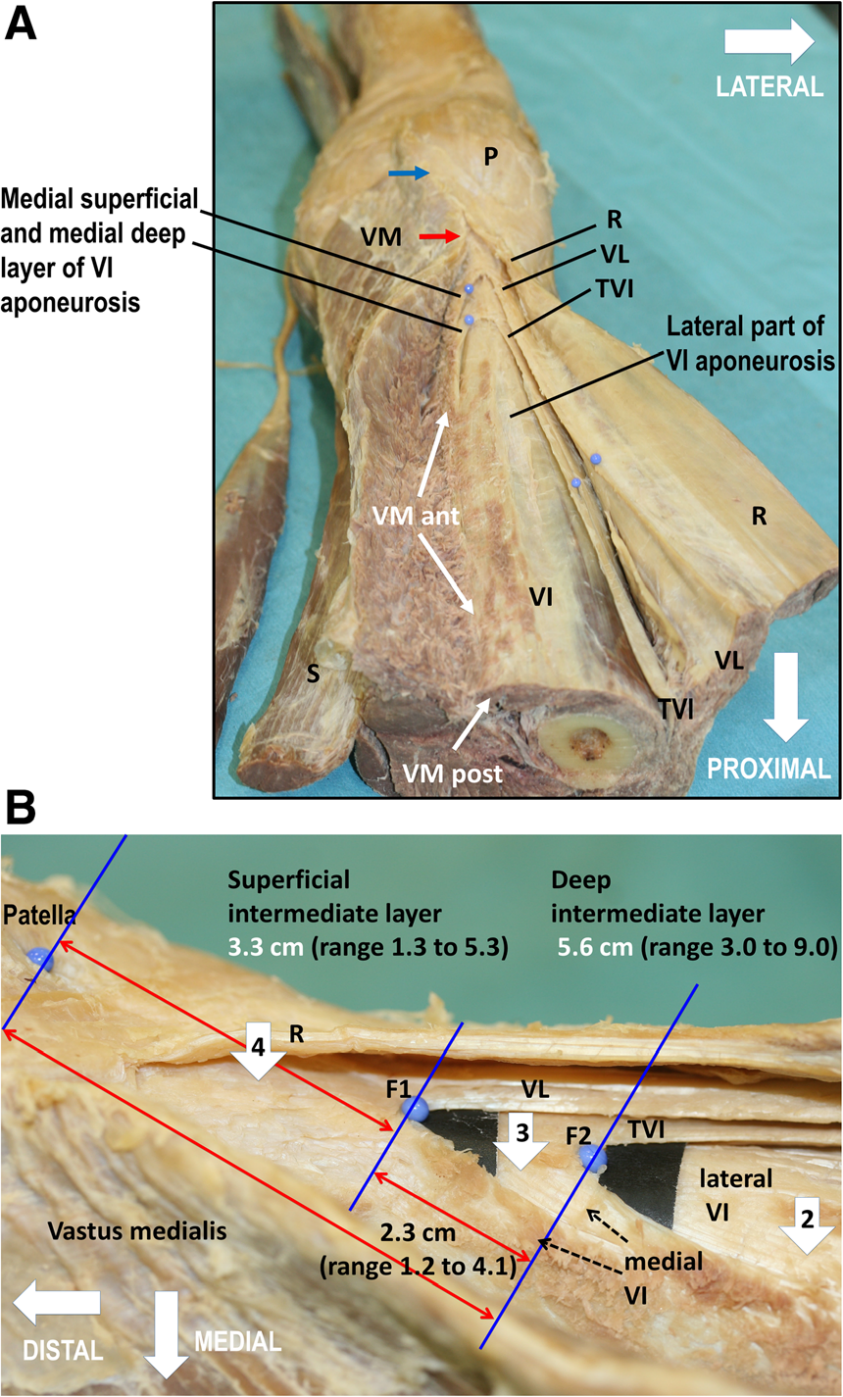
Architecture of the multilayered quadriceps tendon. The distal aspect of a right thigh **a** with corresponding distal section of the quadriceps tendon **b** is shown. **a** The architecture of the quadriceps tendon consisting of the rectus femoris (R), vastus lateralis (VL), tensor vastus intermedius (TVI), vastus intermedius (VI) is shown. The medial part of the VI aponeurosis separates into a superficial and deep medial layer. Sartorius (S), patella (P). The insertions of the vastus medialis (VM) into the patella and capsule of the knee joint (*blue arrow*), rectus femoris (*red arrow*) and vastus intermedius (*green arrow*) is marked. Anterior insertion of the vastus medialis in the the vastus intermedius (VM ant), posterior insertion of the vastus medialis into the vastus intermedius (VM post). **b** The proximal two blue dots (F1 and F2) mark the fusing points of the intermediate layer of the quadriceps tendon. All portions of the extensor apparatus fuse over a region ranging from 13 to 90 mm proximal to the superior pole of the patella (*distal blue dot*). Lateral portions of the vastus intermedius (lateral VI) form the deepest layer of the quadriceps tendon. The superficial and deep layer of the medial vastus intermedius aponeurosis (*black dotted arrows*) fuse 56 mm (range, 30 to 90 mm) and 33 cm (range, 13 to 53 mm) proximal to the patella with the aponeurosis of the tensor vastus intermedius (TVI) and vastus lateralis (VL) respectively. Together they built the two-layered intermediate layer of the quadriceps tendon. The tendon of the rectus femoris (R) forms the superficial layer of the quadriceps tendon. For better visualization the fusing point are underlined with black paper. The vastus medialis is released from its insertion into the vastus intermedius and rectus femoris. Depending on the level of virtual transection one finds two, three or four layers (*white arrows with numbers*). An oblique transection could lead to the impression of a complex multi-layered arrangement of the quadriceps tendon

**Fig. 4:**
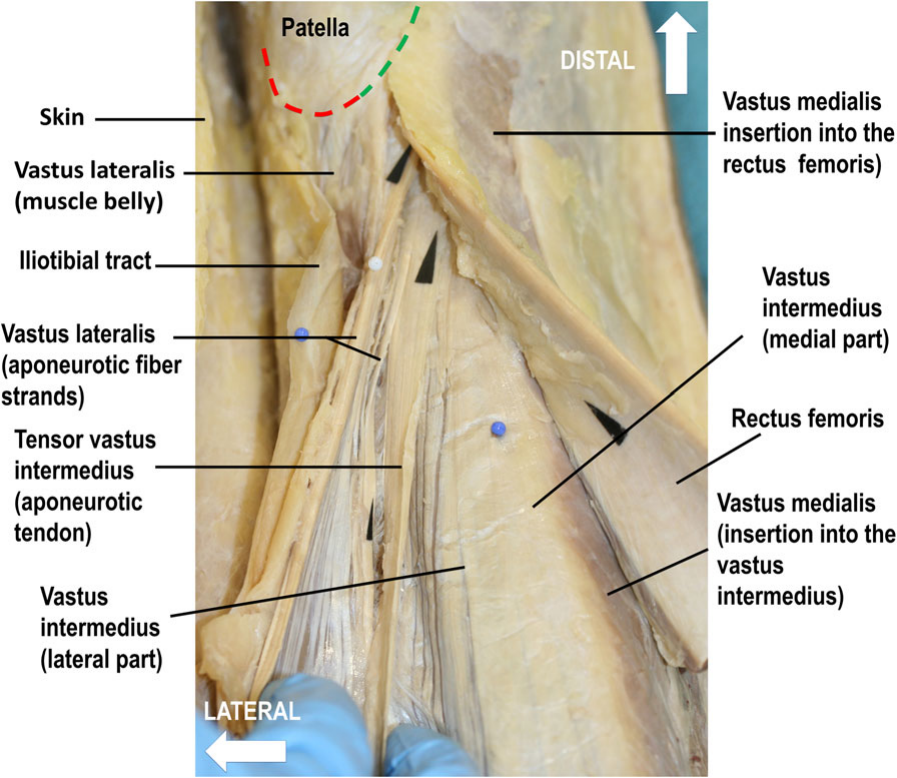
Distal aspect of the extensor apparatus of the knee joint above the patella. The rectus femoris muscle is reflected medially displaying the anterior insertion of the vastus medialis into the vastus intermedius (see also Fig. 3a). The vastus medialis further inserts into the rectus femoris and patella. The medial deep and medial superficial layer of the vastus intermedius aponeurosis are covered by the inserting muscle fibers of the vastus medialis and therefore not visible. The aponeurosis of the vastus lateralis is separated into two fiber bundles. The fibers of the vastus lateralis and the tensor vastus intermedius are oriented towards the medial femoral condyle. Together with parts of rectus tendon the vastus medialis occupies the supero-medial half of the upper semi-circle of the patella (*green dotted line*). The strong muscle belly of the vastus lateralis inserts at the supero-lateral semi-circle of the patella (*red dotted line*)

The tendon of the vastus intermedius showed a complex multi-layered structure. It converged towards the patella and divided into a lateral and medial part. The lateral part of the vastus intermedius aponeurosis formed the deepest layer of the quadriceps tendon. These lateral fibers were oriented towards the medial femoral condyle (Fig. 2). The medial part of the vastus intermedius aponeurosis separated into a superficial and deep medial layer with an orientation towards the lateral femoral condyle (Figs. 2 and 3a). The fibers of the deep medial vastus intermedius aponeurosis were located above the lateral part of the vastus intermedius aponeurosis. Five to 10 mm medial to the mid-line of the quadriceps tendon, the superficial and deep layer of the medial vastus intermedius aponeurosis fused with the aponeurosis of the tensor vastus intermedius and vastus lateralis respectively. Together they formed the two-layered intermediate layer of the quadriceps tendon. Therefore, the vastus intermedius contributed first to the deep layer of the quadriceps tendon through its lateral part of the vastus intermedius aponeurosis and second to the two-layerd intermediate layer of the quadriceps tendon by its superficial and deep medial VI aponeurosis (Fig. 3). Jointly the different fibers continued towards the patella. The fusion point of the fibers for the deep intermediate layer was on average 56 mm (range, 30 to 90 mm, SD +/− 21) proximal to the patella (Fig. 3b). In the distal aspect the superficial medial layer of the vastus intermedius aponeurosis turned into a tendinous gliding layer of the vastus medialis. The latter, in turn, extended to the medial proximal margin of the patella, and in the deeper aspect to the tendon of the rectus femoris. The superficial medial layer of the vastus intermedius aponeurosis that fused with the aponeurosis of the vastus lateralis (superficial intermediate layer) met 33 mm (range, 13 to 53 mm, SD +/− 14) above the patella (Fig. 3b). The fibers of the vastus lateralis were often composed of bundles of individual thin fiber strands (Fig. 4). The meeting point of the superficial intermediate layer was always distal (23 mm, range, 12 to 41 mm, SD +/− 0.9) to the meeting point of the deep intermediate layer (Fig. 3b).

The superficial layer of the quadriceps tendon was formed by the tendon of the rectus femoris. Proximal to the meeting points of the two-layered intermediate layer, the aponeurosis of the rectus femoris was located directly on the lateral part of the vastus intermedius aponeurosis (= deep layer of the quadriceps tendon). In other words, 56 mm (range, 30 to 90 mm) proximal to the superior border of the patella, an intermediate layer was missing (Table 1). Therefore, at this site the quadriceps tendon was seen to be composed of two layers only, separated by thin partitions of fat. In contrast, distal to the meeting points of the two-layered intermediate layer, the quadriceps tendon was composed of four layers (Fig. 3b). The various layers of the quadriceps tendon were joined to each other through light, divisible crosswise fibers. The latter had also inlets of fatty tissue to a differing extent. Distally the superficial medial vastus intermedius aponeurosis and the deep gliding aponeurosis of the vastus medialis fused with the tendon of the rectus femoris and the patella. Together with parts of rectus femoris tendon the vastus medialis occupied the supero-medial half of the upper semi-circle of the patella (Fig. 4). Remainder of the aponeurosis of the rectus femoris continued superficial to the patella to join the patellar ligament.

**Table 1:** Fusion points of the two-layered intermediate layer of the quadriceps tendon

Case Nr.	side	Deep intermediate layer (mm)	Superficial intermediate layer (mm)	Distances between the fusion points (mm)
1	right	30	18	12
2	left	45	30	15
3	left	72	53	19
4	right	90	49	41
5	left	81	46	35
6	right	59	36	23
7	right	66	41	25
8	right	46	25	21
9	left	34	19	15
10	right	34	13	21
mean		55.7	33	22.7
max		90	53	41
min		30	13	12

Table 1 indicates the individual data (*n* = 10) of the fusion points (distances to the patella in millimeters) of the two-layered intermediate layer of the quadriceps tendon. The medial deep layer of the vastus intermedius aponeurosis fused with the aponeurosis of the tensor vastus intermedius (*deep intermediate layer*). The medial superficial layer of the vastus intermedius aponeurosis fused with the aponeurosis of the vastus lateralis (*superficial intermediate layer*). The meeting point of the superficial intermediate layer was always distal (23 mm, range 12 to 41 mm, SD +/− 0.9) to the meeting point of the deep intermediate layer. All elements of the quadriceps tendon fused over a region proximal to the patella ranging from 13 to 90 mm (Fig. 3b)

In three cases the superficial intermediate layer separated from the deep intermediate layer more proximal. The former again fused further distally with the deep gliding aponeurosis of the vastus medialis and the tendon of the rectus femoris.

The strong muscle belly of the vastus lateralis inserted at the supero-lateral semi-circle of the patella (Fig. 4). In a fully extended knee joint, fibers of the lateral components of the extensor apparatus were oriented towards the medial superior border of the patella and subsequently towards the medial femoral condyle. Fibers of the medial components of the extensor apparatus were oriented towards the lateral superior border of the patella and subsequently towards the lateral femoral condyle (Figs. 2, 3 and 4).

**Fig. 5:**
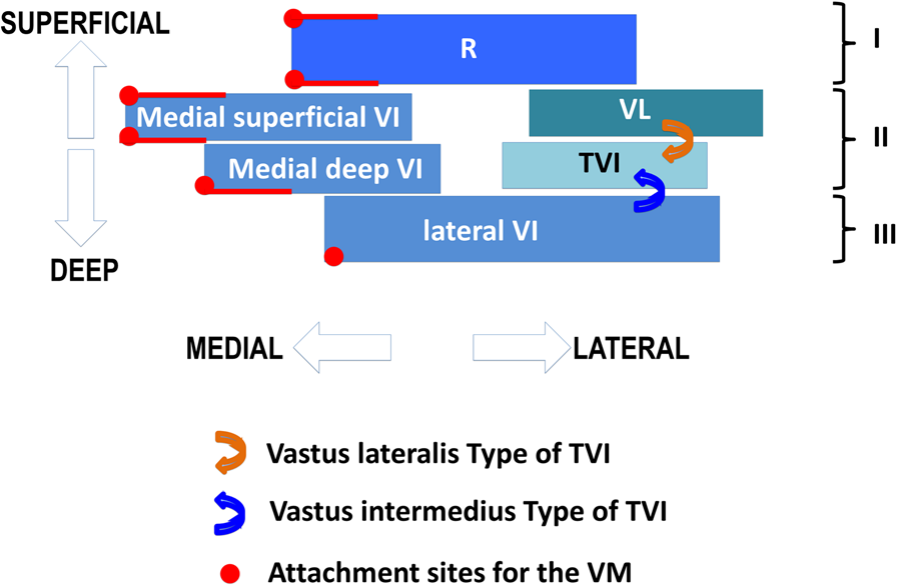
Schematic drawing of the three-layered architecture of the quadriceps tendon. The superficial layer (I) of the quadriceps tendon is formed by the tendon of the rectus femoris (R). The intermediate layer (II) is further sub-divided. The tendon of the vastus intermedius (VI) itself shows a complex multi-layered structure consisting of the lateral part of the vastus intermedius aponeurosis (lateral VI) and the medial deep and medial superficial layers of the vastus intermedius aponeurosis. The medial superficial and the medial deep layer of the vastus intermedius aponeurosis fuse with the aponeurosis of the tensor vastus intermedius (TVI) and vastus lateralis (VL), respectively. The lateral part of the vastus intermedius aponeurosis forms the deepest layer (III) of the quadriceps tendon. In some cases, the aponeurosis of the tensor vastus intermedius emerges either from the lateral part of the vastus inermedius (*bended blue arrow*) or vastus lateralis (*bended orange arrow*) (Grob et al.). The vastus medialis (VM) is not directly involved in the architecture of the quadriceps tendon. It inserts into the aponeurosis of the vastus intermedius and tendon of the rectus femoris on its anterior and posterior side (*indicated by the red dots and lines*)

In four cases an independent and strong aponeurosis of the tensor vastus intermedius was found (*n* = 4 independent type) (Grob et al.). In one case the aponeurosis of the tensor vastus intermedius was rather weak and greater portions of the lateral vastus intermedius aponeurosis divided into two layers. An identical pattern was found in three cases where the aponeurosis of the tensor vastus intermedius emerged from the lateral part of the vastus intermedius (*n* = 4 vastus intermedius type) (Grob et al.). In these situations, the anterior part of the lateral vastus intermedius aponeurosis fused with the deep medial vastus intermedius aponeurosis forming the deep intermediate layer of the quadriceps tendon. In two other cases the aponeurosis of the tensor vastus intermedius arose from the vastus lateralis (*n* = 2 vastus lateralis type) (Grob et al.). Thus the vastus lateralis contributed to both layers of the intermediate layer of the quadriceps tendon (Fig. 5).

## Discussion

Published data about the structure of the quadriceps tendon are diverse. While some authors observed three layers (Andrikoula et al. 2006; Iriuchishima et al. 2012; Sonin et al. 1995; Warwick & Williams 1973; Yablon et al. 2014), others report two, three or more layers (Waligora et al. 2009; Zeiss et al. 1992). Anatomy textbooks often give no special attention to the structure of the quadriceps tendon and state briefly that the four muscular elements of the quadriceps muscle group fuse in the quadriceps tendon (Moore et al. 2014; Netter 2011; Pabst 2008; Platzer 2013; Schünke et al. 2011). In the present dissections a consistent tri-laminar structure of the quadriceps tendon was found. However, the intermediate layer could be further sub-divided. Besides this description of the laminar organization the present findings provide information about the fiber orientation and the insertion of the different components of the extensor apparatus of the knee joint into the patella.

The present study differs from traditional anatomic descriptions in as much as it adds the tensor vastus intermedius to the architecture of the extensor apparatus (Grob et al.; Rajasekaran & Hall 2016).

Our dissections revealed that the fibers of the aponeurosis of tensor vastus intermedius have their own position in the deep intermediate layer of the quadriceps tendon and insert into the medial superior border of the patella (Fig. 3).

Similar to previous reports (Andrikoula et al. 2006; Iriuchishima et al. 2012; Sonin et al. 1995; Waligora et al. 2009; Warwick & Williams 1973; Yablon et al. 2014; Zeiss et al. 1992) the present study found variations in the structure of the quadriceps tendon. However, the variability was restricted to the fusion point rather than the number or structure of the individual layers (Fig. 3).

In four cases an independent aponeurosis of the tensor vastus intermedius could be traced. In five cases the aponeurosis of the tensor vastus intermedius either arose from the vastus intermedius or the vastus lateralis. In one case an independent but weak aponeurosis of the tensor vastus intermedius was observed. However, these variations did not change the general architecture of the quadriceps tendon.

In contrast to textbooks of anatomy (Moore et al. 2014; Netter 2011; Pabst 2008; Platzer 2013; Schünke et al. 2011) we found a two-layered medial aponeurosis of the vastus intermedius. Thus, it contributed to the deepest as well as to the intermediate layer of the quadriceps tendon. The vastus medialis, as an important dynamic stabiliser against laterally directed forces, inserted in all layers of the vastus intermedius aponeurosis (Figs. 2, 3a and 5). Hence, not only the vastus medialis, but also the vastus intermedius represents a dynamic restraint to lateral tracking of the patella. In contrast to some publications (Andrikoula et al. 2006; Iriuchishima et al. 2012; Sonin et al. 1995; Warwick & Williams 1973; Yablon et al. 2014) the vastus medialis is not directly involved in the architecture of the quadriceps tendon. It inserts into the aponeurosis of the vastus intermedius and tendon of the rectus femoris on its anterior and posterior side.

We postulate six elements of the multi-layered quadriceps tendon based on the current dissection (Fig. 5): Lateral aponeurosis of the vastus intermedius, deep and superficial medial aponeurosis of the vastus intermedius, vastus lateralis, tensor vastus intermedius and rectus femoris. All these elements – with differences in fiber direction - join each other a certain distance proximal to the patella (Fig. 3). Despite the complex structure of the quadriceps tendon and individual differences its anatomic arrangement is well structured.

The situation becomes complex and confusing when the quadriceps tendon is viewed at different cross sections. There is a high variability regarding the fusing point of the superficial and deep intermediate layer (between 13 and 90 mm proximal to the superior base of the patella). This and the oblique orientation of the two-layered intermediate layer appear to be the major reasons for the published diversity of the architecture of the quadriceps tendon (Waligora et al. 2009; Zeiss et al. 1992). Depending on the level of transection or MRI cut one finds two, three or four layers (Fig. 3b). Additionally, depending on the direction of the plane the corresponding layers can be complete or incomplete. An oblique transection or MRI cut could easily lead to the impression of a complex multi-layered arrangement of the quadriceps tendon. Furthermore, the aponeurosis of the vastus lateralis, can separate into two or three fiber bundles (Fig. 4) causing additional confusion. Zeiss et al. studied the MRI appearance of 52 knees with normal tendons. They described that the interpretation of the architecture of the quadriceps tendon is especially difficult in its intermediate layer. They found that the number of laminations was variable, with either two (30 %), three (56 %) or four layers (6 %). In 8 % of the knees, the laminations were barely visible (Zeiss et al. 1992).

In contrast to previous investigations, the present study traced all components of the extensor apparatus from the origin to insertion (Figs. 1 and 3). This enabled us to outline the different layers over the whole expansion of the muscle components. The architecture of the quadriceps tendon based on cross- and longitudinal transections (Waligora et al. 2009; Zeiss et al. 1992) is limited and makes an interpretation difficult or even impossible.

The components of the extensor apparatus are arranged like the layers of an onion or the “layered husk of corn” (Fig. 1). A similar view of the anatomy was expressed as early as 1912 by Poirier (Poirier & Charpy 1912).

The medial and lateral muscle fibers of the extensor apparatus lie opposite each other and join 5 to 10 mm medial to the mid-line of the tendon. This arrangement and its orientation towards the medial and lateral femoral condyles support the view that medial and lateral forces of the quadriceps muscle group balance each other. The vastus lateralis, the tensor vastus intermedius and the lateral part of the vastus intermedius counterbalance the medial parts of the vastus intermedius (superficial and deep layer) and the inserting vastus medialis. The rectus femoris also predominantly inserts into the medial aspect of the superior border of the patella. The vastus intermedius and rectus femoris provide an extensive area for the attachment of the vastus medialis (Fig. 3a).

A quadriceps tendon graft may be used to reconstruct the anterior cruciate ligament (Crall & Gilmer 2015; Geib et al. 2009; Lee et al. 2016; Lee et al. 2007; Marshall et al. 1979; Slone et al. 2015), the posterior cruciate ligament (Chen et al.; Chen et al. 2004; Wu et al. 2007), the medial patellofemoral ligament (Lenschow et al. 2015; Steiner et al. 2006), the lateral collateral ligament (Chen et al. 2001) and the Achilles tendon (Arriaza et al. 2016). This autograft shares biological and mechanical properties with other grafts such as the patellar ligament or hamstrings, sometimes with superiority (Han et al. 2008). Harvesting the quadriceps tendon (with or without patellar bone) might have an impact on the function of the extensor apparatus of the knee joint as a whole. The removal of a tendon graft probably alters the delicate interplay between different layers of the extensor apparatus. Chen et al. reported that 9 % of subjects exhibited donor site pain after quadriceps graft harvesting, and the risk of occult partial rupture of the remaining quadriceps tendon may exist. Late quadriceps tendon rupture at the donor site following harvesting of a quadriceps tendon graft has been reported (Pandey et al. 2015). Loss of quadriceps muscle strength of 20 % after harvesting the quadriceps tendon graft for anterior cruciate ligament reconstruction and prolonged weakness of knee extension strength, predominantly in women, have also been reported (Chen et al. 2006; Yasuda et al.). However, others report low donor-site morbidity when using a quadriceps tendon graft compared to a bone tendon bone graft of the patellar ligament (Gorschewsky et al. 2007; Han et al. 2008; Lund et al. 2014). The harvesting technique may also impact the outcome. For example, if the quadriceps tendon is harvested at the fusing points (Fig. 3b) it is questionable that such a graft is suitable as a firm graft. A harvest of the quadriceps tendon medial to the fusing points of the intermediate layers violates the insertion of the vastus medialis with potential consequences on the terminal phase of extension and patellar stability (Lieb & Perry 1971; Pocock 1963; Toumi et al. 2007). On the other hand a lateral harvest of the quadriceps tendon compromises the insertion of the vastus lateralis and the tensor vastus intermedius. Based on the present anatomic findings it can be assumed that a harvest of a tendon graft lateral of the fusing points of the two-layered intermediate layer would be of better quality than a medial graft removal (Fig. 3a). Questions arise whether a partial- or full-thickness graft should be harvested and how closure of tendon defects should be performed. Latter questions also arise with regards to parapatellar approaches to the knee joint.

## Conclusion

In conclusion, the findings of the present study revealed a complex but constant architecture of a three-layered quadriceps tendon which is formed by six elements. These are 1. lateral aponeurosis of the vastus intermedius, 2. deep and 3. superficial medial aponeurosis of the vastus intermedius, 4. vastus lateralis, 5. tensor vastus intermedius and 6. rectus femoris. These elements of the extensor apparatus join each other proximal to the patella in a complex onion-like architecture. Its two-layered intermediate layer shows variable fusions points. The vastus medialis contributes to the quadriceps tendon with its medial insertion into all layers of the quadriceps tendon. Further studies are needed to translate the anatomical findings into clinical relevance in patellofemoral pathology or knee surgery.

Our study has few limitations. Inter individual differences between specimens’ height were not considered in the present study. An other important limitation is that the quadriceps tendon was investigated in embalmed cadaveric specimens from elderly donors. Age-related muscle atrophy may well have distorted some results. In addition, embalmed tissue has been reported to shrink by 2.2 to 12 % (Cutts 1988; Friederich & Brand 1990). This could have affected absolute values for variables such as measured fusing points of each layer, their distance to the patella and the distances between the fusing points of the quadriceps tendon and therefore are not likely to be representative of normal healthy adults. Nevertheless, the fundamental architecture of the quadriceps muscle group is likely to have been preserved. Considering the complexity of the quadriceps tendon further investigation of its morphology in healthy young individuals is warranted.
